# 

**Published:** 1872-03

**Authors:** 


### Books and Pamphlets Received.

A Treatise on Human Physiology, designed for the use of students and practitioners of medicine. By John C. Dalton, M.D. Fifth edition revised and enlarged, with two hundred and eighty-four illustrations. Philadelphia: H. C. Lea, 1871. Buffalo: Thedore Butler & Son.

An Introduction to Pathology and Morbid Anatomy. By T. Henry Green, M.D., London. Illustrated by numerous engravings. Philadelphia: Henry C. Lea, 1871. Buffalo: Theodore Butler & Son.

Physiology of the Soul and Instinct as distinguished from Materialism. By Martyn Paine, A.M., M.D., LL.D. New York: Harper Bros., 1872.

Pulmonary Consumption, its nature, varieties, and treatment. With an analysis of one thousand cases to exemplify its duration. By C. J. B. Williams, M.D., F.R.S., and Charles Theodore Williams, M.A., M.D., Oxon. Philadelphia: Henry C. Lea & Co., 1871. Buffalo: Theodore Butler & Son.

Parturition without pain, a code of directions for escaping from the primal curse. By. M. L. Holbrook, M.D. Second edition. New York: Wood & Holbrook, 1871.

Plain Talk about Insanity, its causes, forms and symptoms, and the treatment of mental diseases. With remarks on Hospitals and Asylums, and the Medico-Legal aspect of insanity. By T. W. Fisher, M.D. Boston: Alex. Moore, 1872. Buffalo: Breed, Lent & Co.

Animal and Vegetable Parasites of the Human Skin and Hair. By B. Joy Jeffries, A.M., M.D. Boston: Alex. Moore, 1872. Buffalo: Breed, Lent & Co.

Annual Report of the Comptroller of the State of New York, transmitted January 2d, 1872.

The Transactions of the American Medical Association. Vol. XXII.

Reports of the Trustees and Superintendent of the Butler Hospital for the Insane at Providence, R. I., January 24th, 1872.

Introductory Lecture to the Course of 1871-'72, in the Medical Department of the University of New York. By Alfred L. Loomis, M.D., Professor of the Institutes and Practice of Medicine.

The influence of Speculative Beliefs in Medicine. By L. C. McElroy, M.D. Re-print from Cincinnati Lancet and Observer, January, 1872.

Electricity in the Treatment of Diseases of the Skin. By Geo. M. Beard, M.D. Reprint from American Journal of Siphilography and Dermatology, January, 1872.

Transactions of the Medical Society of the State of West Virginia.

Twelfth Annual Report of the Superintendent of the State Lunatic Asylum for Insane Criminals at Auburn, N.Y., for year ending Sept. 30, 1871.

Sixteenth Annual Report of the Trustees of the State Lunatic Hospital at Northampton, Mass.

Third Annual Report of the Trustees of the Willard Asylum for the Insane for the year 1871.

First Special Report of the Chicago Relief and Aid Society, Chicago, 1871.

Report of the Board of Health and Board of Consulting Physicians of Lowell, Mass.

Circular of the State Normal School and College at Buffalo, N.Y.

Transactions of the Iowa State Medical Society.

Proceedings of the second Meeting of the American Association for the cure of Inebriates, held in New York, Nov. 14th and 15th, 1871.

Report of the Resident Physician of Brigham Hall Hospital for the Insane, Canandaigua, N. Y., for the year 1871.

Remarks on Medical Ethics; an address to the graduates of Geneva Medical College. By Prof. Charles E. Rider, M. D.

Eating and Drinking. By Geo. M. Beard, M.D. New York: G. P. Putnam & Sons, 1871.

Annual Report of the Superintendent and Physician of the New York State Inebriate Asylum, Binghampton, N.Y., for 1871.

Fourth Annual Report of the Albany City Dispensary Association, October 3d. 1871.

Fourth Annual Report of the New York Orthopædic Dispensary.

The Detection of Criminal Abortion and a study of Fœticidal Drugs. By Ely Van De Warker, M.D., Syracuse, N.Y.

A Plea for Scientific Reforms, a letter to Rev. Theodore L. Cuyler, D.D. By Geo. M. Beard, M. D., New York, 1872.

Small Pox: the predisposing conditions and their prevention. By Dr. Carl Both. Boston: Alex. Moore, 1872. Buffalo: Breed, Lent & Co.

Report of the Board of State Commissioners of Public Charities. Relating to the Insane and the cost of the several State Insane Asylums. Transmitted to the Legislature March 9, 1871.

The Question of Quarentine: The nature and prevention of communicable Zymotic diseases. A paper read before the Medical Library and Journal Association of New York, Jan. 5, 1872. By Alfred L. Carroll, M.D.

First Annual Report of the Board of Managers of the Buffalo State Asylum for the Insane. Presented to the Legislature January, 1872.

